# Evaluating documentation of social history in paediatric medical notes at a regional paediatric centre – a quality improvement initiative

**DOI:** 10.1186/s12887-020-02395-0

**Published:** 2020-11-03

**Authors:** DJ Foley, JK Hannon, CS O’Gorman, AM Murphy

**Affiliations:** 1 Department of Paediatrics, University Hospital Limerick, Dooradoyle, Limerick, Ireland; 2 School of Medicine, University of Limerick, Limerick, Ireland

**Keywords:** Social, History, Paediatrics, Documentation

## Abstract

**Background:**

A child’s home and family environment plays a vital role in neuro-cognitive and emotional development. Assessment of a child’s home environment and social circumstances is an crucial part of holistic Paediatric assessment.

**Aims:**

Our aim is to achieve full compliance with comprehensive documentation of biopsychosocial history, for all children medically admitted to the children’s inpatient unit in University Hospital Limerick.

**Methods:**

We performed a retrospective chart review to audit documentation within our department. This was followed by teaching interventions and a survey on knowledge, attitudes and behaviour of paediatric non-consultant hospital doctors (NCHDs) towards the social history. We performed two subsequent re-audits to assess response to our interventions, and provided educational sessions to seek improvement in quality of care.

**Results:**

Results showed a significant improvement in quality of documentation following interventions, demonstrated by a net increase of 53% in levels of documentation of some social history on first re-audit. Though this was not maintained at an optimum level throughout the course of the year with compliance reduced from 95% to 82.5% on second re-audit, there was nonetheless a sustained improvement from our baseline. Our qualitative survey suggested further initiatives and educational tools that may be helpful in supporting the ongoing optimisation of the quality of documentation of social history in our paediatric department.

**Conclusion:**

We hope this quality improvement initiative will ultimately lead to sustained improvements in the quality of patient-centred care, and early identification and intervention for children at risk in our community.

## Background

Research has shown that a child’s home and family environment plays a vital role in neuro-cognitive and emotional development [1]. The interplay between ecology, biology and child health and development is vital in holistic paediatric healthcare. Much of the fundamentals of child health can be improved by interventions focusing on physiological, socio-environmental, and health and well-being measures. The original Marmot review published in 2010 highlighted the discrepancies in health outcomes created by social inequalities, and was quintessential in describing the effect of social factors on health -, that is to say the lower a person’s social position, the worse the resulting health outcomes are [2]. This review made six crucial policy objectives, including giving every child the best start in life, enabling all children and adolescents to achieve their potential and maximising control over their lives [2]. To do so, they recommend ensuring high quality maternity care and early childhood education, improving resilience amongst children, obtaining equality in resources that optimise emotional and physical development and improving access to education and learning of skills that will improve quality of life [3]. The document ‘Health Equity in England – The Marmot Review 10 Years On’, revisits progress made on recommendations made in the original paper. Children born in deprived areas are more likely to suffer serious illness or long-term disability, and poorer educational outcomes [4]. Disturbingly, health outcomes in some areas have declined in the last decade (from 2010 to 2020), despite increased awareness of the importance of social determinants of health (SDH). Life expectancy has fallen in some areas of London and there has been damage made to gains in health and wellbeing in Scotland, Wales and Northern Ireland [4]. A number of factors including reduced government spending, increasing child poverty, unaffordable housing, and zero hour contracts, have resulted in this stagnation in social improvements in several European countries [4]. Heightened awareness of social inequity has also been reflected in a review by Paediatric Research discussing the impact of SDH on various health conditions and outcomes [5].

Ireland has the highest birth rate in the European Union (12.6 per 1,000 population) and 21.1% of the population is under 15 years of age [6][7]. University Hospital Limerick serves a catchment area of over 100,000 children less than 16 years of age, many from diverse ethnicities and socio-economic backgrounds. In our hospital catchment area, the ‘Mid-West’ of Ireland, 24.7% of the population are classified as living in a disadvantaged or extremely disadvantaged area, and within Limerick City itself, 40% of the population are classified as living in a disadvantaged area [8]. This level of socio-economic deprivation may affect our epidemiology in comparison to other areas, and certainly highlights the importance of the social history as part of medical admission documentation.

Documentation of social determinants can vary significantly from institution to institution and may be affected by presence of paper chart versus electronic healthcare record (EHR). Even for those institutions with electronic records, narrative physician notes may produce additional information from those coded within the EHR [9]. The details of social history documented change depending on the age of the patient (from infant to adulthood), and using prompts within an EHR may result in more complete data records [10]. Some countries within the European Union have designated sections within EHRs in which social determinants can be documented, thus assisting with collection of this data and flagging social issues which may otherwise go overlooked [11].

## Aims

Our aim is to achieve 100% compliance with comprehensive documentation of biopsychosocial history of all children on admission to the children’s inpatient unit in Limerick University Hospital.

Our objective is to assess the quality of documentation of biopsychosocial history at point of admission of medically admitted paediatric patients. We will analyse this information, compare it with national and international paediatric standards, and will present the results to our paediatric colleagues. We will then provide teaching sessions and quality improvement initiatives to improve standards of care.

## Methods

A random sample of 75 patient charts were reviewed over a three-week period from August to September 2019, with equal numbers audited and randomly selected from both paediatric inpatient wards (infant and toddler ward and children’s ward). Patient charts selected included both acute admissions and elective transfers. Medical paediatric charts only were included – patient charts were excluded if they were admitted under a surgical speciality. Audit data was collected in an anonymous fashion using a standardized data collection tool from the paediatric medical admission note, and data analysis was performed manually. Data was stored in line with GDPR (General Data Protection Regulation) requirements.

Standards used were those set by the Health Service Executive (HSE), providing guidance on documentation for medical admission entries. This is available as an online resource titled ‘HSE Standards and Recommended Practices for Healthcare Records Management’ (May 2014) for use in all patients [12]. Another HSE document titled ‘Integrated Care Guidance: A practical guide to discharge and transfer from hospital’ also supports the thorough documentation of social history as part of admission documentation and best practice care for our patients [13]. These documents highlight the importance of the social history as part of medical assessment and admission documentation, and that it is part of the HSE standards and best practice to do so.

Data points collected on documentation of paediatric social history included but were not limited to information on housing, household constituents, ethnicity, parental literacy, school attendance and support services. Following the results of this audit we completed the ‘Plan, Do, Study, Act’ (PDSA) cycle. We analysed this data and presented the results to our paediatric colleagues in a constructive learning environment, as a reflection of current practice. Two teaching sessions were provided to medical staff within our paediatric unit following this audit during our monthly departmental audit meeting sessions. These teaching interventions involved presentations with results of audit and re-audits to date, our progress in documentation, and areas requiring improvement. We held interactive open group discussions on quality improvement, and received feedback from NCHD colleagues on barriers to documentation of social history and relevant challenges faced in practice. Each session lasted one-hour in duration. Feedback was received from senior colleagues within the department during these sessions on how to improve quality of documentation, and various tools which may aid our practice. Posters with memory aids were introduced in the emergency department and on the inpatient wards targeting those clerking new admissions. Introduction of a formal pro forma document for social history was discussed but deemed to be not feasible during this time, due to external factors. We then re-audited our practice four months later, with review of 20 patient charts, using the same data collection tool.

**Fig. 1:**
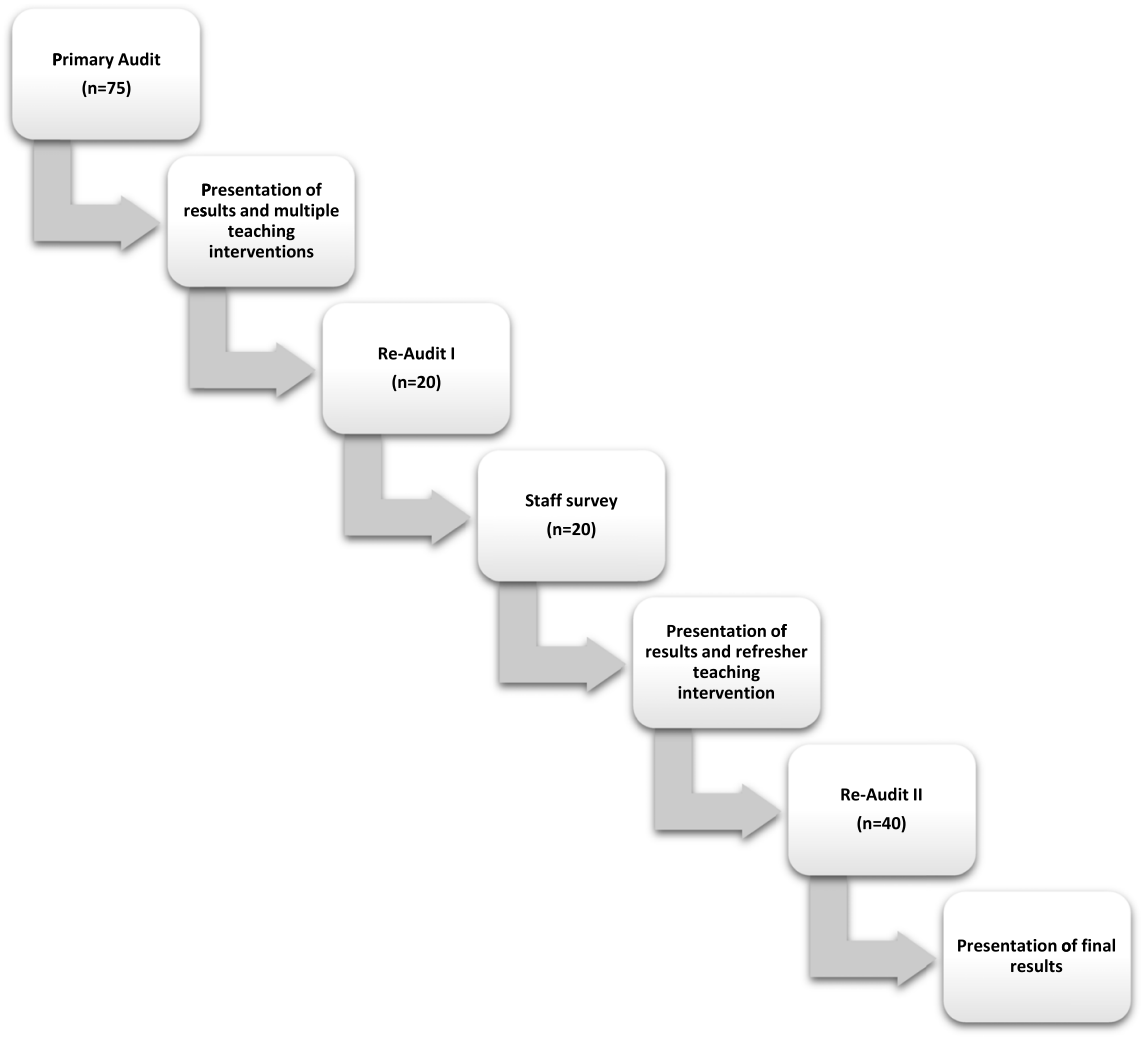
Study methods

We performed an anonymous staff survey to assess knowledge, attitudes and behaviour towards documentation of the social history. This included collection of qualitative data volunteered by NCHDs on subjective barriers to documentation of a Paediatric social history. The survey included 11 questions – ten related to frequency of, and factors and conditions affecting an NCHD’s likelihood of taking a social history, and one free-text question enquiring about subjective opinions on barriers affecting documentation of social history. We provided one further interactive ‘refresher’ teaching session to medical staff within our paediatric unit following this re-audit and presentation of results. We subsequently performed a final re-audit of 40 patients and presented these results to our department. See summary of study methods in Fig. 1, below.

## Results

### A Primary audit

**Fig. 2:**
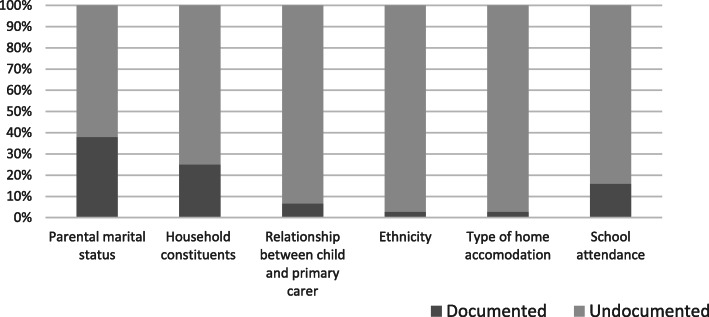
Primary Audit – documentation of constituents of social history.

Documentation of any social history occurred in 31 of 75 charts analysed (41%). The most common areas documented were parental marital status and household constituents. Areas which may have important implications for disease progression, include smoking status and type of home accommodation were recorded in two (2.6%) and zero (0%) charts respectively. Parental occupation was documented in two (2.6%), presence of pets in the home in one (1.3%) and parental literacy in zero (0%) cases (see Fig. 2 below).

### B Re-audit I

**Fig. 3:**
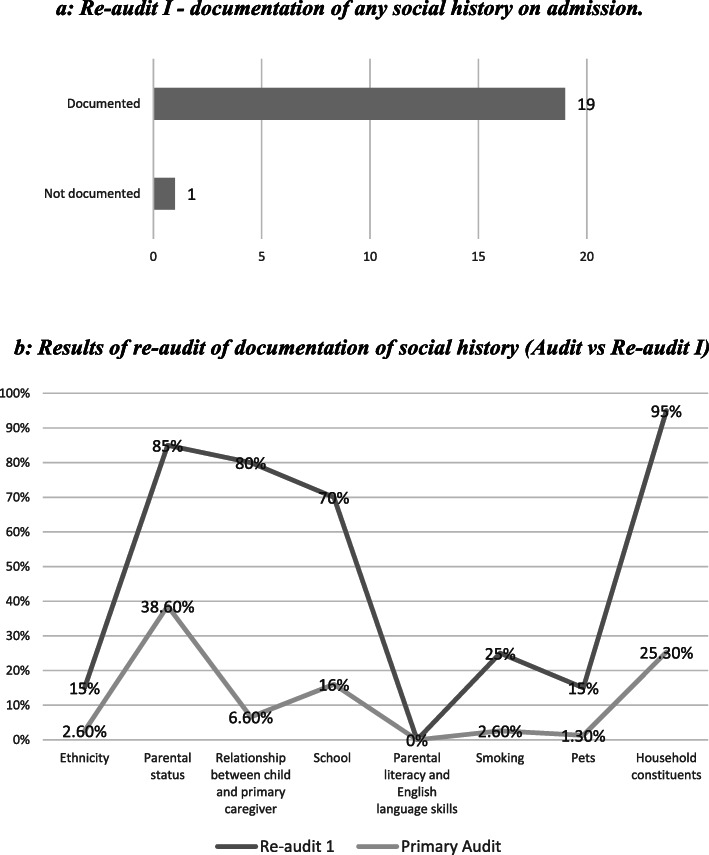
**a**: Re-audit I - documentation of any social history on admission. **b**: Results of re-audit of documentation of social history (Audit vs Re-audit I)

Some improvements were noted on the first re-audit on documentation of social history. Most notably, the percentage of patients with any social history documented increased from 41.3 to 95% of patients. This translates to an improvement of 230% on the original rate, and net increase of 53.7% (Fig. 3a below). Figure 3b also demonstrates documentation of various aspects of the paediatric social history.

### C Staff survey

This survey was released at two timepoints. It was first circulated following re-audit I, and was then re-released once again several months later prior to re-audit II, to target those who did not complete the survey on the first instance. The questions remained the same and each paediatric doctor filled the survey just once. A total of 13 non-consultant hospital doctors (three interns, six senior house officers and four registrars) completed the survey.

NCHDs self-reported that they attempted to take a social history ‘always’ or ‘often’ 84.6% of the time. They were most likely to take a social history in the emergency department (53.8%), followed by the inpatient paediatric ward (38.5%) and least likely to obtain social history in the outpatient department (7.7%). They felt more likely to take a social history for an emergency (76.9%) than elective (23.1%) admission. Participants felt more likely to document a social history between normal daytime working hours of 9am and 5pm (84.6%). Fatigue and increased workload negatively affected ability to document social history.

**Fig. 4:**
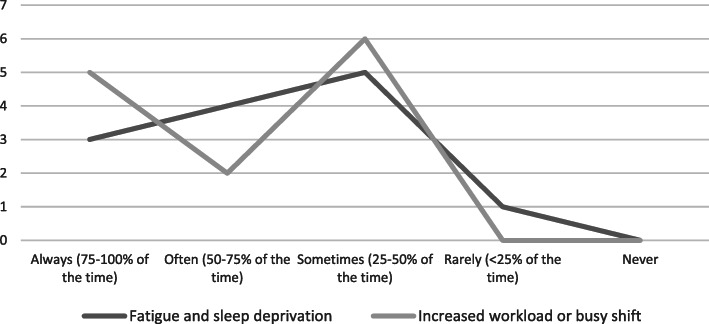
Survey results - Barriers to documentation of a social history. ‘How often are the following factors felt to negatively affect NCHDs ability to document a social history?’

NCHDs felt more likely to omit a social history in the emergency department if they felt the child was not going to require admission to the inpatient ward. 38.5% of NCHDs felt that they often or sometimes forget to document a social history, in contrast with 61.5% who subjectively felt that they rarely or never forgot to document this. Other factors discouraging our NCHDs from taking a social history include feeling ‘nosy’, ‘embarrassed’ or that they may ‘offend’ parents, or indeed feeling that the environment is not appropriate to ask such confidential information. Symptoms of fatigue and sleep deprivation, and an increased workload were subjectively felt to negatively affect the NCHDs ability to complete a social history most of the time (demonstrated in Fig. 4 below).

It is worth noting that our daily morning handover meeting routinely follows the ISBAR format (‘Identify, Situation, Background, Assessment, Recommendation’) to optimise clear and concise handover of relevant details and maintain good timekeeping. A concise handover of information is key, especially during the busy winter months. Therefore details of a child’s social history are generally only discussed if deemed to be a highly important part of the child’s admission, for example this may include patients admitted with alcohol or substance misuse or in adolescents or admission under Sect. 12 of the Childcare Act (1991) for child safety concerns [14]. It is otherwise discussed in further detail during the ward round. Qualitative feedback within our survey by some NCHD’s that the social history was felt to be perceived by senior consultant colleagues and the NCHDs themselves to be unimportant in comparison with medical information. Several doctors felt that feedback of social history was for the most part ‘underestimated by NCHDs’ and that senior colleagues ‘did not pay attention [to social history]’.

### D Re-audit II

**Fig. 5:**
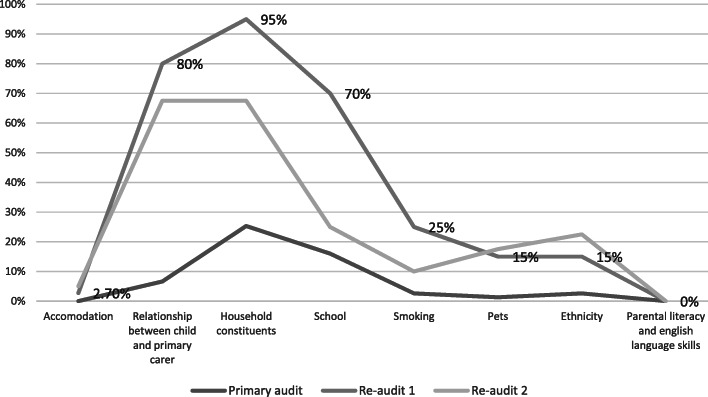
Constituents of social history as documented in primary audit and re-audits I and II.

33 of 40 patients (82.5%) had some element of social history documented in the second re-audit. This is a reduction from 95% on results of previous re-audit. Household constituents and relationship of the child to the primary carer were most likely to be documented – both 67.5% of the time. School attendance was noted in 25% of children – although in those under the age of four years old, primary school attendance is not applicable due to age. In Ireland, children are entitled to free pre-schooling from approximately two years eight months old and onwards. In this study, one child was documented as attending pre-school. Parental literacy, language skills and respite or additional social supports were not documented in any cases. Parental smoking status and presence or absence of pets in the home was documented in 10% and 17.5% respectively. Figure 5 below outlines results of primary audit and re-audits I and II.

## Discussion

The 2016 Irish census demonstrated that a higher proportion of children are now living in rented accommodation and in flats and apartments, leaving families somewhat more vulnerable than those in an ‘owned’ home [7]. Housing instability including homelessness, frequent changes in accommodation and arrears in rent can result in adverse outcomes. Fifteen-hundred families in Ireland are homeless, including over 3,300 children [15]. This figure does not include hidden homelessness, where families are relying on family and friends to provide temporary accommodation.

### The family unit and housing instability

Lone parents and children of lone parents are particularly at-risk groups for social deprivation. These groups have a persistently higher absolute social risk gap in income poverty in comparison with their counterparts in the European Union (EU). This cohort accounts for 27% of Irish children [16]. As a result, these children in lone parent families and those living with disability are much more vulnerable to poverty and deprivation compared to traditional family units and those without disability [16].

Adverse childhood experiences (ACEs) can lead to increased rates of common childhood conditions such as asthma, headaches and allergies and poorer self-reported health [17]. ACEs may include neglect or environmental stressors such as living with substance abuse or domestic violence. The added burden of these co-morbid childhood illnesses may ultimately result in increased school absenteeism and long term negative impacts on education and future economic prospects [17].

### Alcohol, tobacco and illicit drug use

Alcohol use in adolescents has become increasingly common over the last two decades. Most presentations of adolescents with acute intoxication tend to occur at weekends and during holiday periods [18]. We are increasingly seeing the use of alcohol in combination with other illicit substances e.g., marijuana and 3,4-methylenedioxy-methamphetamine (MDMA), and its use may act as a gateway to facilitate progression on to other illicit drugs. A recent Croatian study noted that there was co-morbid drug use in 6.25% of adolescents presenting to their hospital with acute alcohol intoxication [18]. Alcohol can also act as a risk factor for overweight and obesity [19]. Heavy alcohol use in parents may increase prevalence of childhood and adolescent consumption of alcohol, and associated negative outcomes [20].

Though tobacco use in adolescence and in adulthood has declined in recent years, there has been a startling jump in e-cigarette use in adolescence [21]. Maternal smoking may result in reduced lung function in adulthood, and exposure to parental smoking can trigger respiratory infection during the childhood years [22].

Prenatal marijuana exposure may cause attention deficit and hyperactivity symptoms in childhood and adolescence [23]. Marijuana exposure may cause severe neurological signs including ataxia, hypotonia, and respiratory compromise in young children [24]. Chronic exposure to marijuana in childhood and adolescence can cause permanent neurocognitive damage, psychological morbidity and psychiatric illness [25]. There may be increased exposure to marijuana in children living in areas where recreational and medical use of the substance has been legalised [25, 26]. A systematic review of sport and drug use in adolescence found that engagement in sporting activities during adolescent years may be a protective factor in preventing use of illicit drugs [27].

### Child safe-guarding and parental mental health

Social history is a crucial component of care in assessment of the mother and new-born infant’s readiness for discharge from hospital to the community. As this is a particularly vulnerable time for both parties it is vital that the maternal and infant clinical status, social supports and follow-up care plan are appropriate for discharge [28]. Education should be adequately completed and any added vulnerabilities safe-guarded [28]. Increased psychological distress in mothers has been noted to be a risk factor for Emergency Department presentation within the first year of life, emphasising the need to fully assess the psycho-social situation in the home before discharge [29].

Social history is a key measure in early identification of families who may have vulnerabilities to or ongoing child abuse. By assessing strengths and weaknesses in a child’s environment and identifying potential harms, we may prevent or effectively manage many more cases of childhood physical, sexual, or emotional abuse, and neglect. One notable intervention is the Safe Environment for Every Kid (SEEK) model [30, 31], introduced as a trial in paediatric primary care in the United States. This programme successfully improved rates of addressing depression, substance abuse, intimate partner violence, stress and healthcare worker comfort level with up to 36 month from time of training [31].

Intimate partner violence is a significant risk factor for child abuse. A study performed by et al. showed that those who were violent with their partners were more likely to use violence against children [32]. In a study performed in the United States (US) 64% of parents reported physical disciplinary measures with their children aged 19–35 months of age. Early ‘spanking’ has been shown to be associated with poor cognitive development in early childhood and there is a link with aggression, school-age behavioural problems, and increased risk of physical abuse [33].

### Diet and Lifestyle

Many adult diseases can root in early childhood from risk factors such as unhealthy eating habits, poor education and exposure to maltreatment [1]. Toxic stress in early childhood can produce physiological changes to the developing brain, immune system and metabolism, which may persist into the adult years [34]. As socioeconomic status increases, the trend to healthy weight and distribution of body fat, and improved cardiorespiratory fitness also increases [34]. Children who grow up in lower-socioeconomic status households tend to have poorer health in adulthood, irrespective of their own adult socio-economic status [34]. Exposure to adverse environments in childhood and in adulthood may contribute to increased mortality from cardiovascular disease and some malignancies, in those from lower socio-economic groups living in more deprived areas [35].

Lower income households have also been shown to have more missed school days and limitation of activities related to asthma control [36].

### Ethnicity

Research to date has highlighted the various risks of certain diseases amongst different ethnicities.. Background genetic risks are compounded by social determinants of health and barriers in access to education and care to produce poorer health outcomes [37]. Socio-economic status also acts as a significant mediator in race and ethnicity survival association for various childhood malignancies [38]. In provision of healthcare, families may feel pressured to conform to Western rituals which differ from their own belief system and values. The cultural beliefs of a family with regard to healthcare provision and end of life care should be explored in a sensitive manner, and the integrity of these customs and provisions should be preserved [39]. It is also important to note that migrant children and adolescents have higher rates of suicidal ideation and suicide attempts. This again highlights the need for culturally sensitive preventative and intervention measures [40].

Direct provision is one area of particular vulnerability in our social care system. In October 2018 there were over 6,000 people living in direct provision, with nearly 1,800 of these being children [41]. These families live in restrictive environments with many other families and individuals, and without adequate access to kitchen facilities and recreational space. The effect this social exclusion has on children living in this environment can have a dramatic impact on child health and well-being, and higher risk of spread of infectious disease [41]. Most recent reports have shown that approximately 14% of all children living in direct provision have been referred to the child and family agency for social work supports, compared to 1.6% of children in the general population [42].

Overall our study highlights the gaps in documentation of a paediatric social history and the challenges that we face in improving this. Though there was an initial marked improvement with the quality of documentation following multiple teaching interventions, when this was sub-optimally sustained despite provision of refresher sessions.

When qualitative information was sought of our NCHD cohort as to barriers in obtaining a paediatric social history; time constraints, tiredness and sleep deprivation were quoted. Some NCHDs felt that many parents did not understand the reasons for taking a social history and that they therefore felt uncomfortable asking such questions, subsequently deterring doctors further. As discussed previously, there may be an institutional under-estimation of the importance of the social history.

Difficulties in documentation of, and adequate focus on social determinants of health (SDH) in a medical setting have been demonstrated in the literature previously. Barriers may be increased by frustrations due to time constraints and difficulties in accessing resources to address social needs [43]. Medical staff may also find it difficult to adjust to prioritising a non-clinical intervention [43]. A trial on an adult patient populations by Vest et al. suggested the benefits of automated risk-stratified interventions within a healthcare organization may result in increased social work referrals and improved rates of attendance at medical appointments, an approach which may be helpful in paediatric practice [44].

NCHDs suggested that further departmental teaching sessions encouraging documentation of the social history would be important. General feedback from the survey suggested that further teaching sessions may be helpful for all paediatric staff, including confidential discussion of patient cases where the social history was key in identification of socioeconomic risk factors or child safe-guarding, and where social history was key in holistic bio-psycho-social patient management.

Important confounding factors include the changeover of medical staff. Though most doctors remained in the department for the full year (July 2019- July 2020), two registrars, two senior house officers and all of the interns left the department after a 3–6 month rotation. This changeover in staff may affect knowledge of and consistency in documentation reflected in our audit. However we note that 80% of our cohort of registrars remained the same for the 12-month period, and the majority of paediatric admission notes were by a paediatric medical registrar (92% of admissions in our primary audit (69/75)). Another limiting factor affecting our results may be the intercurrent pandemic and its effect on staffing issues, departmental provision of education sessions and progress of quality improvement initiatives.

The use of various tools as memory aids and to trigger reminder of importance of documenting the paediatric social history as part of a child’s medical record may be helped by the use of standardised tools such as IHELLP (Income, Housing, Education, Legal Status, Literacy, Personal Safety). This tool has been shown to significantly improve documentation of the paediatric social history [45]. This tool triggers the interviewer to inquire about income supports, housing, education, legal and immigration status, language and personal safety. This tool was examined in one study of over 600 Paediatric admissions made by 87 doctors. Education sessions were provided to the intervention group prior to commencement of the study. More than 80% of the intervention team documented a social history using the IHELLP aide, and a subsequent three-fold increase in social work consults made by the intervention team in contrast with the control team [46].

## Conclusions

Our job as healthcare professionals is to ensure that patient care needs are best met. National guidelines suggest that by assessing the needs of individuals we must take into account the physical, psychological, social and emotional needs of the person [13]. This will result in effective planning of healthcare that is tailored to the individual, and minimised delays in discharge.

Documentation of social history is vital as a part the patient’s medical record. Understanding a child’s social background is key to optimizing their emotional and physical well-being. The social history is a tool which enables us to do this, and to lead us on a path which may identify risks to the child, and indeed parental frustrations and challenges. It may uncover risks such as sub-optimal home living conditions, exposure to substance misuse, primary carer mental health issues or a gap in service provision. By supporting a child’s home environment and by attempting to understand the various cultural and family dynamics which may apply, we can optimize child health in a holistic and long-lasting way, that will not only benefit their health but the health of the family unit.

## Data Availability

Data generated or analysed during this study are included in this published article, and in its supplementary information files. Further data stored securely in line with GDPR guidelines and available on request.

## Abbreviations

ACE: Adverse Childhood Experience
HER: Electronic Healthcare Record
EU: European Union
GDPR: General Data Protection Regulation
HSE: Health Service Executive
IHELLP: Income, Housing, Education, Legal Status, Literacy, Personal Safety
ISBAR: Identify, Situation, Background, Assessment, Recommendation
MDMA: 3,4-methylenedioxy-methamphetamine
NCHD: Non-Consultant Hospital Doctor
SDH: Social Determinants of Health
SEEK: Safe Environment for Every Kid
US: United States
